# Modeling and Analysis of an Opto-Fluidic Sensor for Lab-on-a-Chip Applications

**DOI:** 10.3390/mi9030134

**Published:** 2018-03-19

**Authors:** Venkatesha Muniswamy, Chaya Bangalore Muniraju, Prasant Kumar Pattnaik, Narayan Krishnaswamy

**Affiliations:** 1 Department of Electronics and Communication Engineering, Sai Vidya Institute of Technology, Bengaluru, Karnataka 560064, India; chaya.bm@saividya.ac.in (C.B.M.); narayank101@gmail.com (N.K.); 2 Department of Electrical Engineering, BITS-Pilani, Hyderabad Campus, Hyderabad, Telangana 500078, India; pkpattnaik@hyderabad.bits-pilani.ac.in

**Keywords:** microfluidic channel, microparticles, fluid flow rate, lab-on-a-chip, SOI waveguide

## Abstract

In this work modeling and analysis of an integrated opto-fluidic sensor, with a focus on achievement of single mode optical confinement and continuous flow of microparticles in the microfluidic channel for lab-on-a-chip (LOC) sensing application is presented. This sensor consists of integrated optical waveguides, microfluidic channel among other integrated optical components. A continuous flow of microparticles in a narrow fluidic channel is achieved by maintaining the two sealed chambers at different temperatures and by maintaining a constant pressure of 1 Pa at the centroid of narrow fluidic channel geometry. The analysis of silicon on insulator (SOI) integrated optical waveguide at an infrared wavelength of 1550 nm for single mode sensing operation is presented. The optical loss is found to be 5.7 × 10^−4^ dB/cm with an effective index of 2.3. The model presented in this work can be effectively used to detect the nature of microparticles and continuous monitoring of pathological parameters for sensing applications.

## 1. Introduction

A lab-on-a-chip (LOC) is a device that integrates many laboratory tests on a common integrated circuit. The size of LOC is in the range of a few millimeters to a few square centimeters [1]. LOCs requires the combination of microfluidics, manipulation and study of microliters of fluids and light-fluid interaction. Most of the LOC fabrication processes were developed on silicon. The demands for cheap and easy production of LOC’s results in a simple technology for the development of polydimethylsiloxane (PDMS), microfluidic devices [2]. LOCs provides application related advantages, such as low fluid volume consumption, faster analysis, quick response times and compactness of the system due to the integration of multiple functions [3]. 

In most of the opto-fluidic sensors used in LOC applications, the sensitivity and accurate test results depends on microfluidics, light-fluid interaction and light guiding properties of optical waveguide. Microfluidics involves behavior, control and manipulation of fluids that are geometrically constrained to a millimeter scale. At small scales (channel size around 100 nm to 500 µm), the comparison between effects of the momentum of a fluid to that of viscosity is given by Reynolds number and its value can become very low. At lower Reynolds number, fluid flow becomes laminar and molecular transportation occurs due to diffusion [4]. Accurate specifications in chemical and physical properties, such as temperature, concentration, and pressure, results in more uniform reaction conditions and more accurate single- or multi-step reactions [5,6]. In this article, the optical waveguides and microfluidic channel are developed by using silicon as guiding medium. Silicon is transparent to infrared light with wavelengths above 1100 nm [7]. In the development of biosensors, the bio-sample flow rate plays a very important role in its sensitivity. The sensitivity of ultrasensitive and selective non-enzyme using copper wires is found to be high at near infrared region [8]. Electrochemical detection of glucose from whole blood using copper wires [9], radio frequency [10], and capillary-based ring resonators [11] is presented with many other sensing mechanisms [12,13,14,15,16,17]. The most of the literature papers is focused on fabrication and characterization of LOC photonic integrated circuits. The focus of this article is on modeling and analysis of opto-fluidic waveguide based sensor on silicon on insulator (SOI) substrate. 

**Figure 1:**
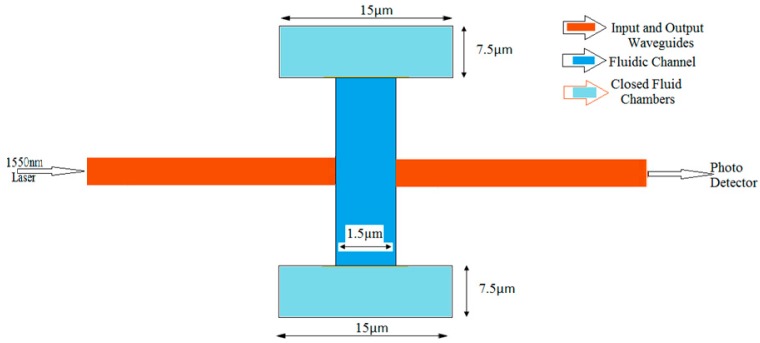
Schematic representation of the integrated biosensor.

Figure 1 shows schematic of an integrated opto-fluidic biosensor employed for modeling and simulation. It consists of a laser source at 1550 nm, photo detector and SOI input and output waveguides (shown in red) with a narrow fluidic channel (shown in blue). In the operation, the optical power is coupled into the input waveguide by using laser source (1550 nm). The guided modes propagate through the input waveguide and couples into the fluidic sensing region. When the light couples from input waveguide to the output waveguide through the fluidic sample the absorption of optical power occurs due to the properties of the microparticles present in the fluidic sample. Based on the fluidic gap distance, a mode mismatch occurs between fluidic gap and input waveguide. Mode mismatch occurs due to the absorption of optical power [18], in fluidic gap. Hence, mode mismatch which occurs during propagation of light through sensing region (fluidic gap distance) and absorption of optical power by the analyte is used for the purpose of analysis and also as a designing tool.

In this article modeling of microfluidic channel to achieve a continuous flow rate between two sealed chambers maintained at different temperatures and pressure acting on the fluidic channel walls is described in Section 2. The modeling and modal analysis of SOI waveguides for use in light propagation is discussed in Section 3. The optical properties of SOI waveguide at infrared wavelength range 1500 nm to 1600 nm and power coupling analysis is depicted in Section 4. 

## 2. Modeling of the Microfluidic Channel

In this section the microfluidic structure shown in blue in Figure 1 is designed and analyzed. Microfluidic channels are small-dimension structures developed to achieve flow rate, sorting, and manipulation of fluids that are geometrically constrained. 

**Figure 2:**
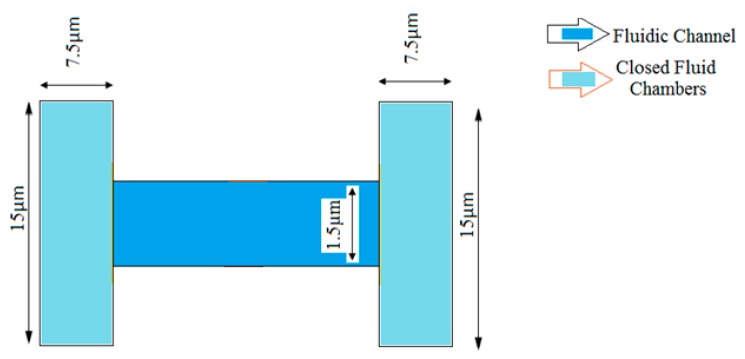
Structure of microfluidic channel.

Figure 2 shows the structure of microfluidic channel used for analyzing the flow rate between the two closed fluidic chambers, which are maintained at different temperatures. It consists of two fluidic chambers having dimensions of 7.5 μm in length and 15 μm in depth. The fluidic chambers are linked by a narrow channel having 1.5 μm width and 15 μm in length. The chamber and channel walls are modeled using silicon as material having a thickness of 1 μm. The walls of the two chambers are in thermal contact with heat sinks maintained at 290 K and 300 K, respectively. The channel walls are thermally insulated. The fluid in the center of the channel is maintained at a pressure of 1 Pa.

The microfluidic channel shown in Figure 2 is used to compute the flow between two sealed chambers (closed fluid chambers) connected by a micro-channel with conducting walls when the chambers are maintained at different temperatures. The material used for the walls is silicon. To achieve a continuous flow rate between two sealed chambers a birefringent object is used with a pressure point at the centroid of channel geometry. For channels of micron-scale dimensions the Knudsen number becomes larger than 0.01. At atmospheric pressure it is, therefore, necessary to use a slip condition on the surfaces of walls in the vicinity of the channel. The slip velocity, *u_slip_*, along the walls of the microfluidic channel is given by Equations (1) and (2) [19]:

(1) $$u_{slip}=\sigma_{s}\frac{\lambda }{\mu }\left(\tau n-\left(\left(n^{T}\tau n\right)n\right)\right)+\sigma_{T}\frac{\mu }{\rho T_{F}}\left[\nabla T_{W}-\left(n\cdot \nabla T_{W}\right)n\right]$$

(2) $$T_{W}=T_{F}-\zeta_{T}\lambda_{n}\cdot \nabla T$$

where *λ* is the mean free path of the fluid, *n* is the boundary normal, *τ* is the viscous stress tensor, *T_W_* is the wall temperature, *T_F_* is the temperature of the fluid, *μ* is its viscosity, and *ρ* is its density. Of the slip coefficients, *σ_s_* is the viscous slip coefficient, *σ_T_* is the thermal slip coefficient, and *ζ_T_* is the temperature jump coefficient can be defined by material properties, *a_v_* is the tangential momentum accommodation coefficient. The slip coefficients are given by Equations (3)–(5) [19]:

(3) $$\sigma_{S}=\frac{2-a_{v}}{a_{v}}$$

(4) $$\sigma_{T}=\frac{3}{4}$$

(5) $$\zeta_{T}=2\frac{2-a_{v}}{a_{v}}\frac{\gamma }{\gamma +1}\frac{\kappa }{\mu C_{P}}$$

where *κ* the thermal conductivity of fluid and the mean free path can be computed from the fluid properties using the following Equations (6) and (7) [20]:

(6) $$\lambda =\frac{1}{C_{O}}\frac{\mu }{\rho \langle c\rangle }$$

(7) $$\langle c\rangle =\sqrt{\left(\frac{8RT}{\pi M_{n}}\right)}=\sqrt{\frac{8p}{\pi \rho }}$$

A continuous flow between the two sealed chambers maintained at slightly different temperatures is achieved by maintaining a pressure of 1 Pa at the centroid of microfluidic geometry. The channel width is 1.5 µm, so the Knudsen number varies from 0.064 and 0.045. 

The relative pressure acting on the channel wall, as a function of position along the wall is represented by mean path of microparticles. As the absolute pressure in a fluid flow is reduced, the mean free path of the fluid molecules begins to approach the size of the vessel through which the flow occurs. The detailed analysis of the velocity magnitude, pressure, and temperature analysis is described in this section.

**Figure 3:**
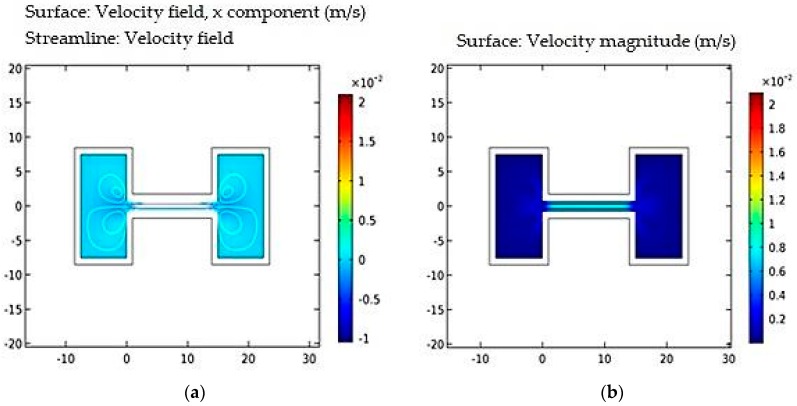
(**a**) Streamline velocity field vs. channel length; and (**b**) velocity magnitude vs. channel length.

Figure 3a, shows a graph of streamline velocity field with respect to channel length. Figure 3b shows a graph of velocity magnitude with respect to channel length. In the steady state there is no net flow through the channel, but a flow parallel to the walls, in the direction of the thermal gradient (cold to hot), develops due to thermal creep. In order to compensate for this flow, a back flow develops in the center of the channel, which is driven by a pressure gradient in the fluid. It results in a continuous flow of fluidic sample in the narrow microfluidic channel. This is achieved by maintaining a constant pressure of 1 Pascal at the center of fluidic channel.

**Figure 4:**
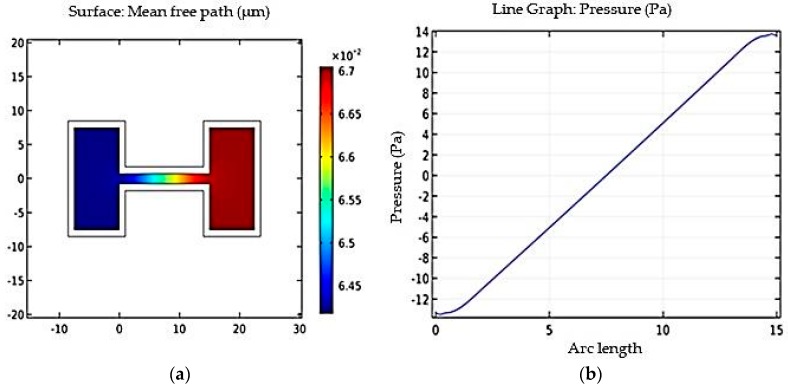
(**a**) Mean free path vs. channel length; and (**b**) pressure along channel length vs. arc length.

Figure 4a, shows fluid mean free path. Such rarefied flows are characterized by a parameter known as the Knudsen number, which is the ratio of the mean free path to the characteristic length of the geometry. Figure 4b, shows the relative pressure acting on the channel wall, as a function of position along the wall.

**Figure 5:**
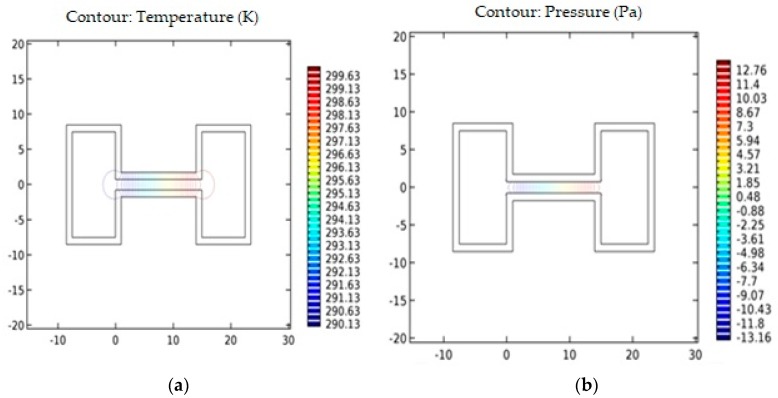
Contour diagram of (**a**) temperature vs. channel length; and (**b**) pressure vs. channel length.

Figure 5a,b shows the temperature and pressure contours within the model. A temperature jump occurs between the vessel walls and the fluid normal heat fluxes occur into the wall from the fluid sample.

The simulation results shown in Figure 3, Figure 4 and Figure 5 are based on quasi-static liquid flow. This type of flow is required in the design of optofluidic sensor where in light fluidic interaction requires quasi-static flow of fluid. Such quasi-static flow enables greater light fluidic interaction and thereby resulting in enhanced interaction time between light and fluid. This results in better sensitivity, which is discussed in Section 4. 

## 3. Modeling of the Silicon on Insulator (SOI) Waveguide 

**Figure 6:**
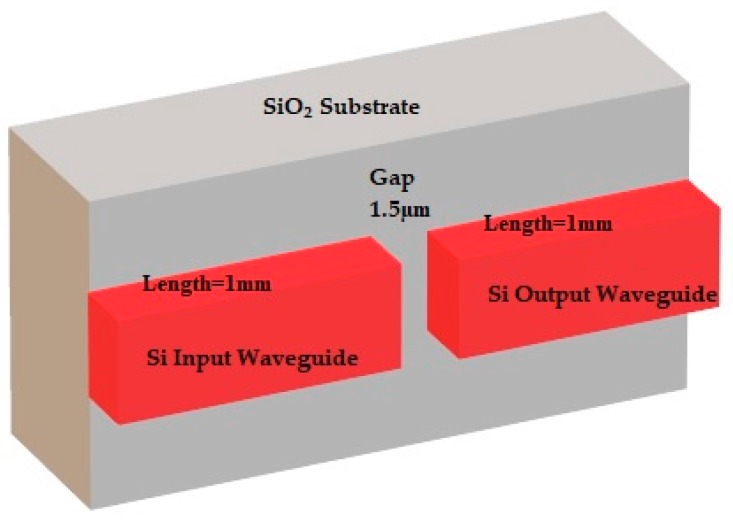
Waveguide geometry.

In this section, the design and modal analysis of a single mode SOI waveguide structure to operate at a wavelength of 1550 nm is presented. The SOI waveguide is designed at infrared region of light spectrum (1500–1600 nm) for single mode operation. Figure 6 shows the geometrical design details of a SOI optical waveguides with gap distance of 1.5 µm. It consists of silicon core, input waveguide and output waveguide having dimensions of 500 nm (waveguide width), height of 250 nm, and length of 1 mm. One of the important parameters used in the design is the cut-off height of the waveguide and is calculated by Equation (8) [21]:

(8) $$h=\frac{\lambda }{2\pi \sqrt{n_{f}^{2}-n_{s}^{2}}}\left[m\pi +\arctan\left(\frac{n_{s}^{2}-n_{c}^{2}}{n_{f}^{2}-n_{s}^{2}}\right)\right]$$

In Equation (8), *h* represents the height of waveguide core, *λ* represents the operating wavelength, *n_s_* is the refractive index of the substrate, *n_f_* is the refractive index of the film, *n_c_* is the refractive index of the cover layer and *m* represents the mode number. For single mode operation, *m* is set to 0 (fundamental mode).

These dimensions results in single mode operation. The substrate is silicon dioxide (SiO_2_), which acts as lower cladding layer for the waveguide. The substrate height is designed for 2 µm and width is designed for 4 µm. 

The dimensions and refractive index of the materials used in the waveguide geometry is shown in Table 1. 

Table 1 shows the refractive index distribution of SOI waveguide (substrate (SiO_2_), core (silicon), and cover layer (air)) at 1550 nm. There is a high refractive index contrast between silicon core (3.3714) and oxide substrate (1.55) index as well as cover layer (air) index. This high refractive index contrast results in SOI waveguides being highly amenable to light guiding at infrared wavelength. This results in excellent propagation characteristics such as dispersion, optical loss and effective index. It is observed that at 1550 nm, single mode operation is achieved with a loss of 5.7 × 10^−4^ dB/cm.

Eigenmode (EM) solver is used for numerically simulate the waveguide geometry which is shown in Figure 6. The simulation settings used in the EM solver is shown in Table 2. Perfectly matched layers (PML) boundary conditions are used in the simulation [22].

**Figure 7:**
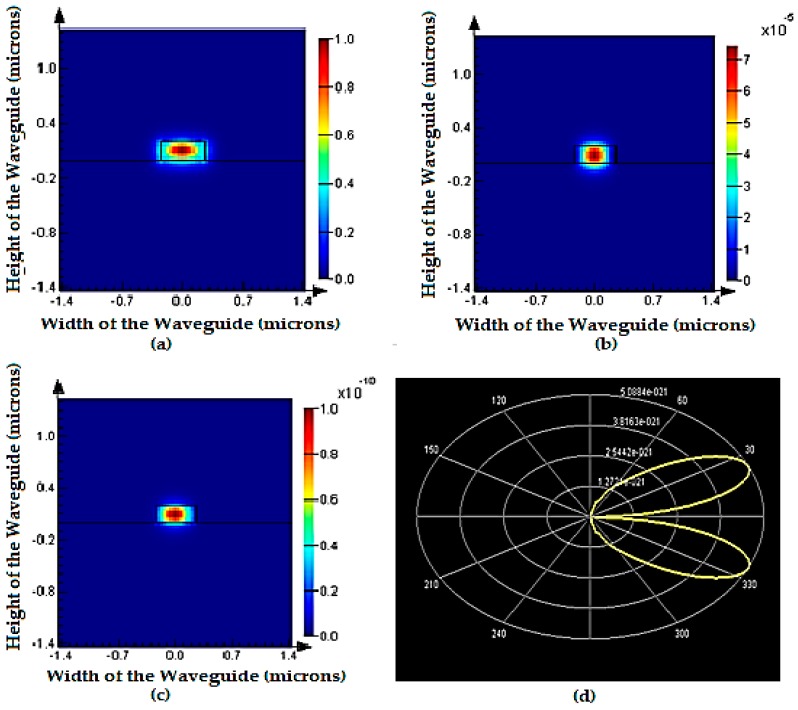
Mode confinement plots at 1550 nm: (**a**) electric field intensity; (**b**) magnetic field intensity; (**c**) energy distribution; and (**d**) polar plot of Poynting vector.

Figure 7 shows the optical mode confinement in SOI waveguide at 1550 nm. It is evident that the optical confinement of light is pronounced at the core of the waveguide (red) when compared to the evanescent wave coupling into the cladding (yellowish-green). Figure 7a,b shows electric field intensity distribution and magnetic field distribution in SOI waveguide. Energy distribution and polar plot of far field vector is shown in Figure 7c,d, respectively. Figure 7d shows the optical power propagation in the core is symmetric along *z*-axis with 90° of half power beam width and there are no side lobes. The absence of side-lobes indicates zero evanescent modes coupling to substrate of the waveguide structure. 

**Figure 8:**
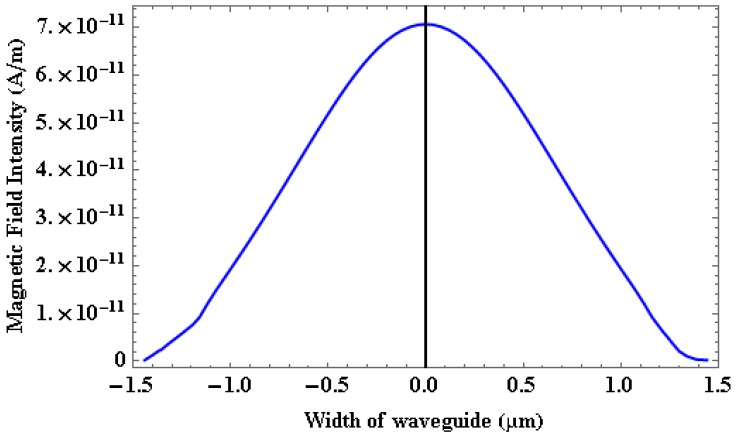
Magnetic field intensity as a function of width of waveguide.

Figure 8 shows the magnetic field (H-field) intensity of SOI waveguide which is confined within the core region of waveguide. With the help of Figure 8, mode field diameter (MFD) is found out to be 1.774 µm for 0.5 µm, waveguide width. Since MFD and width of the waveguide are of similar dimensions indicating very good optical mode confinement.

**Figure 9:**
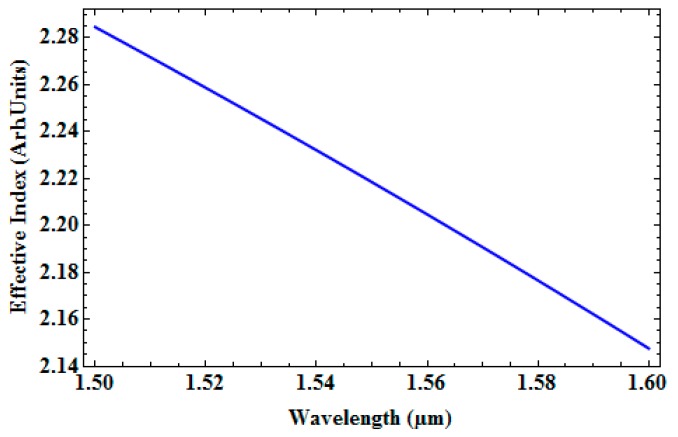
Effective index as a function of wavelength.

**Figure 10:**
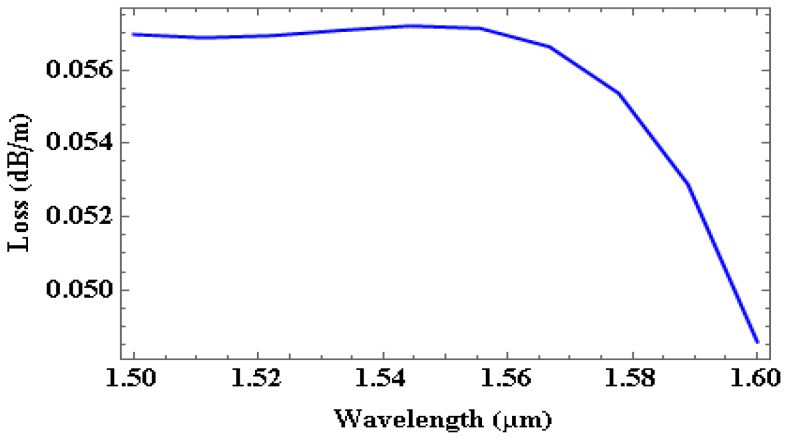
Loss in the waveguide as a function of wavelength.

Figure 9 shows effective index as a function of wavelength and Figure 10 depicts loss as a function of wavelength in SOI waveguide for a wavelength range of 1550 nm to 1600 nm. Table 3 gives the effective index, loss in dB/cm, percentage of TE/TM fraction for the wavelengths 1550 nm, 1530 nm, 1550 nm, and 1600 nm.

**Figure 11:**
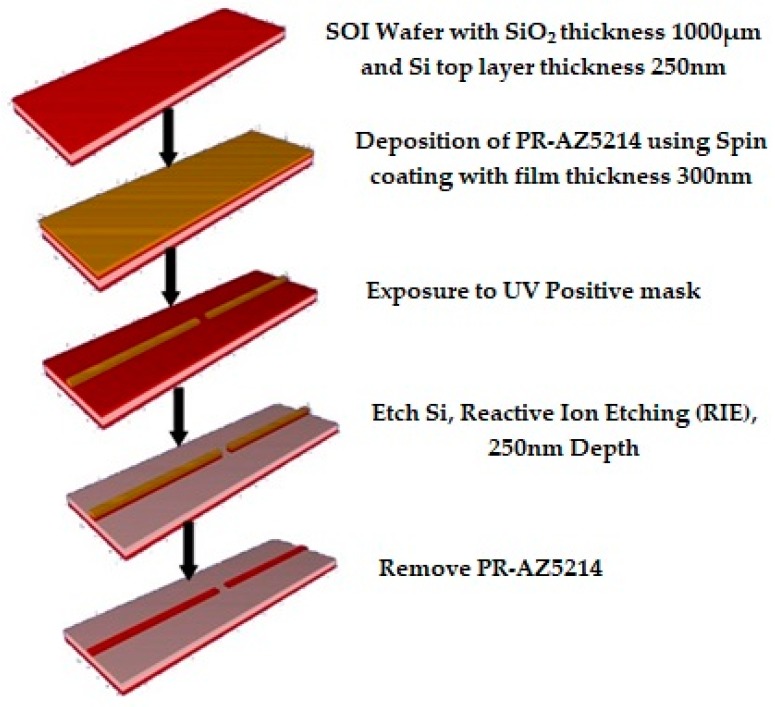
The process flow steps for the silicon on insulator (SOI) waveguide sensor simulated with the Intellisense software package.

The fabrication process flow steps of the SOI waveguide structure has been simulated using Intellisense software (version 8.9, Lynnfield, MA, USA) module and is depicted in Figure 11. 

The fabrication process flow steps includes following steps:

- SOI wafer definition with top device layer (silicon) having a thickness 250 nm and SiO_2_ thickness of 1000 µm.
- Deposition of PR-AZ5214 spin (001) (Microchemicals GmbH, Ulm, Germany) having a thickness of 300 nm. PR-AZ5214 is used as positive mask.
- Lithography ultraviolet (UV) contact: in this step mask is exposed to UV light.
- Etch Si Reactive Ion Etching (RIE) (Cl_2__CF_4_), in this step the UV exposed region of 250 nm thickness, top Si is removed by using reactive ion etching.
- Etch PR-AZ5214 wet (lift-off), in this step PR-AZ5214 is removed by using the lift-off technique.

## 4. Results and Discussion

In this section, power coupling and sensitivity analysis of opto-fluidic sensor shown in Figure 1 is presented. The sensor is designed to operate at 1550 nm. It is observed that for 1500 nm to 1600 nm wavelength range single mode operation is achieved with excellent optical parameters and light confinement in input and output waveguides.

### 4.1. Power Coupling Analysis 

**Figure 12:**
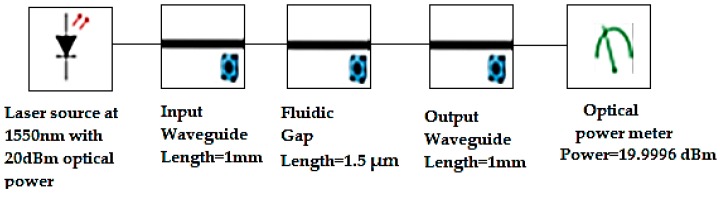
Optical power measurement using Interconnect.

The power coupling simulation analysis has been done using the Lumerical Interconnect module. This simulation analysis has been done to prove the proof of concept and the waveguide design. This simulation setting has been carried for ideal conditions ignoring absorption and dispersion losses. The entire lab-on-a-chip structure as described in schematic representation of Figure 1, has been simulated using 1550 nm laser source with a power level of 20 dBm (100 mw). This analysis has been carried out using Lumerical interconnect simulation tool and schematic arrangement of the structure is depicted in Figure 12. The simulation is carried out by considering the input waveguide, output waveguide with a length of 1 mm, and a fluidic gap distance of 1.5 µm. The total light propagation length is 2.0015 mm. The simulated optical power output is 19.9996 dBm (is only considering mode-mismatch only). This power output is assuming ideal conditions, without any absorption and dispersion losses. The expected theoretical value of output power is (20–0.000114 dBm) 19.9998 dBm, which is calculated using SOI waveguide loss using the data depicted in Figure 10. The simulation arrangement depicted in Figure 12, results in a power output of 19.9996 dBm, which is in close agreement with the theoretical value, 19.9998 dBm. The difference in theoretical and simulated optical output is only 0.0002 dBm. This analysis was carried out to prove the concept proposed in this work. 

### 4.2. Power Coupling and Sensitivity Analysis 

In this analysis absorption by the fluid in the gap is taken into consideration. The amount of light coupled to the output waveguide from the input waveguide (refer Figure 1) depends upon the gap between the waveguides, the normalized power for light coupled between the waveguides is given by Equation (9) [21].

(9) $$P_{nor}=\;\frac{\int_{-\infty }^{\infty }\phi_{1}\left(x\right)\phi_{2}\left(x\right)dx}{\sqrt{\int_{-\infty }^{\infty }\phi_{1}^{2}(x)}\sqrt{\int_{-\infty }^{\infty }\phi_{2}^{2}(x)}}$$

where $\phi_{1}(x)$ is the field (electric or magnetic field) emanating from the input waveguide, $\phi_{2}(x)$ is the field entering the output waveguide (after propagation through the waveguides gap) and *P_nor_* is the normalized power coupling to the output waveguide.

The optical power which is coupled into the input waveguide the guided modes propagates through it and interacts with fluidic sample. Reflection of light takes place at the boundary between silicon core and fluidic sample, as a result of which, the changes occurs in effective index and velocity of light propagating in the fluidic sample. This results in variation of optical power intensity in the sensing region. Most of the light intensity is lost in the sensing region due to absorption of light. The absorption coefficient of the microparticles (hemoglobin) can be calculated using Beers-Lambertz law given by Equation (10) [18]:

(10) $$P_{co}=\;P_{nor}e^{-\varepsilon (\lambda )LC}$$

where *P_co_* is the normalized power coupling to the output waveguide taking into account both mode-mismatch and absorption, *P_nor_* is the normalized power coupling to the output waveguide taking into account mode-mismatch only (as evaluated from Equation (9)), *C* is the molar concentration in moles/liter and *L* is the fluidic path length traversed by the light (optical gap distance). 

The amount of light absorbed in the fluidic gap region is used as a major parameter for determining the sensitivity. At 1550 nm the molar extension coefficient of hemoglobin is almost zero. For sensitivity analysis the, length *L* = 1.5 µm (light fluidic interaction length) is considered with molar concentration of hemoglobin in whole blood is *C* = 150 g/L. The gram molecular weight of hemoglobin is 64,500 g/mole [23]. Sensitivity S of optical sensor can be determined by using (11) [18]. *N* is the effective refractive index:

(11) $$S=\;\frac{\partial P_{co}}{\partial N}$$

**Figure 13:**
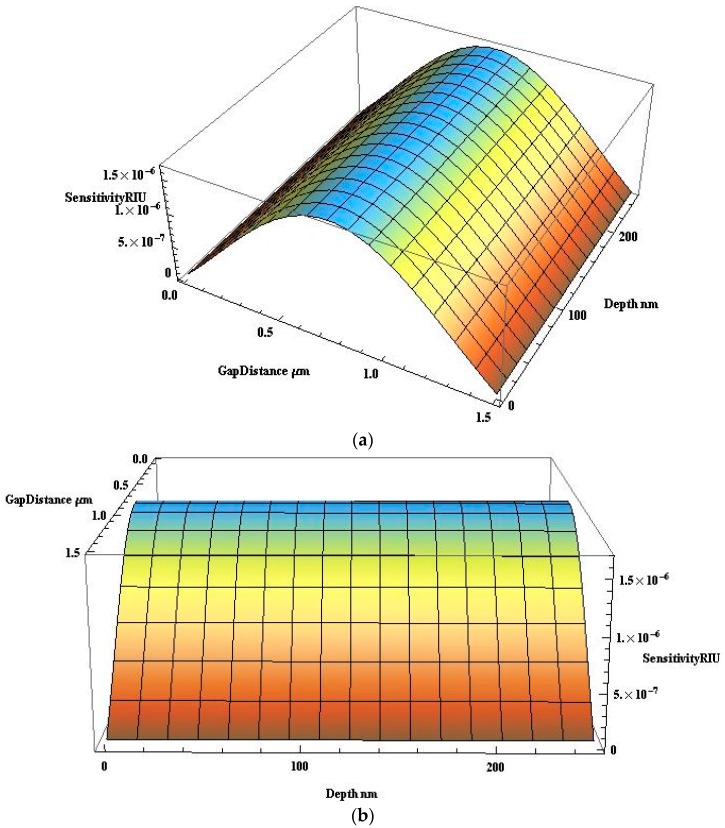
(**a**) Sensitivity in RIU (View 1); and (**b**) sensitivity in RIU (View 2).

Figure 13a,b, depicts two different isometric views of the sensitivity in RIU as function of depth of waveguide and gap distance of the opto-fluidic sensor, the schematic of which is shown in Figure 6. The plot was achieved using Equation (11). 

From Figure 13a,b it may be inferred that sensitivity of up to 1.67 × 10^−6^ RIU can be achieved for a gap distance of 0.8 µm.

The simulation results presented in this work i.e., sensitivity of up to 1.67 × 10^−6^ RIU is in agreement with the experimental work done earlier [24,25,26]. The feasibility of such compact refractometric sensor has been demonstrated by Domachuk et al. [24] wherein highly compact refractometers where integrated along with planar microfluidic geometry for high-resolution refractive index measurements. In the work conducted by S. Mandal et al. [25] a bulk refractive index detection limit of up to 7 × 10^−5^ has been estimated for nano-optical sensor array using refractive index sensing. In the earlier work done by Cole et al. [26] a refractive index detection limit of up to 10^−5^ RIU has been achieved using a waveguide refractometer.

The optimum design parameters of the proposed sensor are listed in Table 4.

## 5. Conclusions

In this work modeling and analysis of integrated waveguides, and micro-fluidic channel for a opto-fluidic lab-on-chip sensor application has been presented. The flow rate analysis between two fluidic chambers connected by a narrow microfluidic channel which is in a plane perpendicular to integrated optical SOI waveguides is presented. The narrow fluidic channel sandwiched between two single SOI waveguides acts as a sensing region. A continuous flow of fluidic sample in the narrow microfluidic channel has been simulated by maintaining pressure of 1 Pascal at the centroid of the fluid channel geometry. The sensor is designed for single mode operation, which, is achieved at 1550 nm for waveguide dimensions of 250 nm (height) and 500 nm (width). The effective refractive index was determined to be 2.3 with a negligible loss of 5.7 × 10^−4^ dB/cm. Sensitivity of up to 1.67 × 10^−6^ RIU can be achieved for a fluidic gap distance of 0.8 µm. The power analysis carried out in this work gives the qualitative measurement of optical properties and nature of the microparticles present in the fluidic sample. Such SOI based opto-fluidic sensors can be effectively used for lab-on-a-chip sensing applications. 

## Figures and Tables

**Table 1:** Materials and dimensions of waveguide.

Material	Width (nm)	Height (nm)	Refractive Index at 1550 nm
Si (Core)	500	250	3.4714
SiO_2_ (Substrate)	4000	1000	1.55

**Table 2:** Eigenmode solver settings.

Parameter	Value
Mesh Resolution	50 × 50
Wavelength	1550 nm
Boundary conditions	Perfectly matched layers (PML)
Background index	1 (Air)

**Table 3:** SOI waveguide parameter.

Wavelength (nm)	Effective Index	Loss in dB/cm	% (TE/TM) Fraction
1500	2.3619	0.00057	73.96/81.6
1530	2.322	0.00057	72.97/81.43
1550	2.2963	0.00057	72.32/81.33

**Table 4:** Design parameters of opto-fluidic sensor.

Parameter	Value
Wavelength	1550 nm
Height of input/output waveguide	250 nm
Width of input/output waveguide	500 nm
Width of fluidic channel	1.5 µm
Length of input/output waveguide	1 mm
Cross section of fluidic chambers	7.5 µm × 15 µm
